# Editorial: Novel Insights Into Female Post-Mating Physiology in Insects

**DOI:** 10.3389/fphys.2022.877222

**Published:** 2022-03-30

**Authors:** Francesca Scolari, Fathiya M. Khamis, Diana Pérez-Staples

**Affiliations:** ^1^Institute of Molecular Genetics (IGM) - CNR “Luigi Luca Cavalli-Sforza”, Pavia, Italy; ^2^International Centre of Insect Physiology and Ecology (icipe), Nairobi, Kenya; ^3^Instituto de Biotecnología y Ecología Aplicada (INBIOTECA), Universidad Veracruzana, Xalapa, Mexico

**Keywords:** reproduction, ejaculate, aging, male accessory glands, behavior, metabolomics, oviposition, germline stem cells (GSCs)

Mating deeply affects female physiology and behavior. The major factors responsible for such post-mating changes that were identified so far comprise the act of mating itself, the ejaculate transferred by the male during copulation, and the female microbiome. The genes regulating post-mating responses have been only partially characterized, and mostly in model organisms. In most insects, the molecules transferred in the male ejaculate are still completely unknown, as are the biosynthetic gene pathways responsible for their production and regulation. Recently, technological advances in the field of omics, three-dimensional analyses of the morphology of the female reproductive tract during and after mating, and high resolution microscopy allowed the tuning of more comprehensive strategies to study insect reproductive physiology, pointing to the establishment of a holistic approach to trace the dynamic processes following mating.

The aim of this Research Topic was to provide an overview of the current knowledge on the multi-faced female post-mating response in insects, also to provide novel targets to be exploited for the control of agricultural pests and disease vectors.

The work by Agudelo et al. focuses on exploring the effects of male age on female post-mating response in the mosquito *Aedes aegypti*. Authors found that increased male age did not impact female fecundity and fertility but instead reduced the ability to prevent female remating, with relevant implications for mosquito population control programs. Moreover, younger males sired more progeny compared to older males, despite both types of males transferred similar numbers of spermatozoa. These findings suggest that male aging differentially impacts the induction of some post-mating changes in *Ae. aegypti* females, further supporting the complexity of these interactions.

Córdova-García et al. show the importance of investigating the function of male accessory glands (MAGs) in the two tephritid pests *Anastrepha ludens* and *Anastrepha obliqua*. Differently from what is known in the medfly, *Ceratitis capitata*, authors showed that the transfer of MAG products alone induced in mated females a similar response to both male pheromone and oviposition host volatiles. This suggests that the behavioral switch in olfactory response from mate to host finding is induced by the whole ejaculate, comprised of both sperm and MAG secretions.

The mini review paper by Scolari et al. describes the studies focusing on the metabolomics analysis of insect reproductive tissues and ejaculates, with particular emphasis given to the methodological approaches available nowadays for this type of analysis and the implications of the characterization of reproductive metabolomes in basic and applied research.

The article by Pogue et al. focuses on shedding light on the reproductive patters in two tephritid fruit flies, *Ceratitis cosyra* and *Ceratitis capitata*. The authors compared fecundity, fertility and patterns of sperm transfer between the two species and found that *C. cosyra* displayed a higher survival and a lower fecundity with respect to the shorter-lived *C. capitata*, although the tendency to remate was similar. This work underlines the diversity between the life histories of these two species and expands our still patchy knowledge on the reproductive biology of *C. cosyra*, a serious threat to agriculture in Africa.

The study by Vyas et al. shows that gravid *Bactrocera dorsalis* females were able to survive and oviposit also after decapitation, suggesting the existence of a brain-independent control of these functions, potentially as a consequence of the relatively decentralized nervous and circulatory system in insects. Such results stimulate further research integrating molecular approaches able to characterize the genes and the related neuronal networks involved in this bypass pathway for oviposition.

The review paper by Hoshino and Niwa provides a comprehensive overview of the current knowledge on the inter-organ relationships regulating the mating-induced female germline stem cells (GSCs) increase. This effect is a recently identified post-mating response in both sexes of the fruit fly *Drosophila melanogaster*. In particular, this review article describes the role of the neuroendocrine system in transmitting mating stimuli to the GSCs in female *Drosophila*.

In conclusion, the contributions to this Research Topic offer different perspectives on the post-mating response in female insects, considering both the effects of male age and seminal fluid secretions, the role of the nervous system in controlling female survival and oviposition, as well as the impact of different life-history traits. The described findings further point at the complexity of this phenomenon and stress the importance of an integrated multidisciplinary approach for its study.

## Author Contributions

FS drafted of the manuscript. DP-S and FMK improved the text. All authors listed have made a substantial, direct, and intellectual contribution to the work and approved it for publication.

## Conflict of Interest

The authors declare that the research was conducted in the absence of any commercial or financial relationships that could be construed as a potential conflict of interest.

## Publisher's Note

All claims expressed in this article are solely those of the authors and do not necessarily represent those of their affiliated organizations, or those of the publisher, the editors and the reviewers. Any product that may be evaluated in this article, or claim that may be made by its manufacturer, is not guaranteed or endorsed by the publisher.

